# La prise en charge psychosociale des contacts de cas confirmés de COVID-19 à Touba, Sénégal

**DOI:** 10.11604/pamj.supp.2020.37.1.25854

**Published:** 2020-09-08

**Authors:** Ibra Diagne, Allé Baba Dieng, Ahmed Sougou, Albert Gautier Ndione

**Affiliations:** 1 Etablissement Public de Santé de Mbour, Thiès, Sénégal,; 2 Centre des Opérations d´Urgence Sanitaire (COUS)/Ministère de la Santé et de l´Action Sociale, Dakar, Sénégal,; 3 Equipe Mobile d´Intervention et de Soutien Psychosocial (EMIS), Dakar, Sénégal,; 4 Centre Régional de Recherche et de Formation à la Prise en Charge du VIH/SIDA (CRCF), Dakar, Sénégal

**Keywords:** COVID-19, sujets contacts, prise en charge psychosociale, Sénégal, COVID-19, contact subjects, psychosocial care, Senegal

## Abstract

Le Sénégal, à l´instar de bon nombre de pays dans le monde, fait face depuis le 02 mars 2020 à la pandémie de la COVID-19. La prise en charge psychosociale des personnes victimes de cet événement inattendu et potentiellement mortel est indispensable. Dès l´enregistrement des premiers cas au Sénégal avec l´annonce du premier cluster dans la localité de Touba, à 191 km de Dakar, le 12 mars 2020, les autorités sanitaires du pays ont mis en place sur les lieux une équipe pluridisciplinaire avec une cellule psychosociale opérationnelle. Cette cellule a mis en place pour une centaine de victimes directes et indirectes des soins immédiats et post-immédiats individuels et/ou en groupes avec des visites à domicile. Au-delà de l´aspect thérapeutique et de soutien de la prise en charge psychosociale de ces victimes de la COVID-19, cette intervention a permis au niveau décisionnel d´avoir le feedback du terrain sur certaines actions qui posaient plus de problèmes qu´elles n´en résolvaient. Le travail psychosocial de terrain a permis une modélisation et un ajustement des interventions dans un contexte particulier de déni de la population locale.

## Abstract (en)

Senegal, like many countries in the world, has been facing the COVID-19 pandemic since March 2, 2020. Psychosocial care for people who are victims of this unexpected and potentially fatal event is essential. As soon as the first cases were registered in Senegal with the announcement of the first cluster in the town of Touba, 150km from Dakar, on March 12, 2020, the country's health authorities set up a multidisciplinary team on the spot with a cell operational psychosocial. This unit has set up for a hundred direct and indirect victims immediate and post-immediate individual and/or group care with home visits. Beyond the therapeutic and support aspect of the psychosocial care of these victims of COVID-19, this intervention allowed the decision-making level to have feedback from the field on certain actions that posed more problems than they did not resolve. The psychosocial field work made it possible to model and adjust the interventions in a particular context of denial by the local population.

## Commentary

Le monde est confronté à la pandémie de la COVID-19 apparue en République populaire de Chine en décembre 2019. L´Organisation mondiale de la santé (OMS) a qualifié la COVID-19 d´urgence de santé publique de portée internationale et la considère comme une pandémie [1]. Le Sénégal, a l´instar de bon nombre de pays, fait face depuis le 02 mars 2020 à cette pandémie. L´ensemble des quatorze régions administratives du pays ont eu à enregistrer des cas positifs. Dans le cadre de la riposte contre ce fléau mondial, selon la stratégie nationale définie [2], les personnes contacts d´un cas confirmé de la COVID-19 sont habituellement isolées et confinées Au Sénégal, en un moment de la riposte, les autorités politico-sanitaires avaient décidés de confinés les personnes contacts au niveau des réceptifs classiques (hôtel, centres d´hébergement, etc.) des régions respectives. Mais ce dispositif diffère d´une localité et à une autre. Dans la ville de Touba, il n´existe pas d´hôtel, ni de centre d´hébergement, alors les cas contacts sont confinés à domicile. Cette pandémie sans précédent entraine des risques psychosociaux et probablement des retentissements sur la santé mentale et le bien-être chez certaines victimes. D´abord du fait que la maladie en question confronte le sujet au réel de la mort et ensuite du fait des mesures de confinement mises par les autorités politico-sanitaires, pour contenir la transmission interhumaine, qui peuvent être mal vécues par certaines personnes [3]. Devant tous ces constats, la prise en charge psychosociale des victimes de la COVID-19 prend toute son importance dans la riposte et dans la prévention des traumatismes psychoaffectifs pouvant découlés de cette affection. C´est dans ce cadre que des équipes mobiles d´intervention et de soutien (EMIS) psychosocial ont été dépêchés dans certaines localités du pays, comme la ville de Touba, à environ 191 Km de la capitale sénégalaise, afin d´y assurer un accompagnement psychosocial global. L´équipe psychosociale de Touba avait pour missions: la prise en charge médicopsychologique des cas positifs, le soutien psychosocial des cas contacts confinés à domicile et le soutien psychologique des intervenants de terrain. Il s´agit d´une toute première expérience de travail psychosocial de terrain autour de la COVID-19 au Sénégal. La peur et l´inexpérience par rapport à une maladie nouvelle très contagieuse caractérisent le contexte. L´objectif de ce travail était d´identifier les difficultés liées au vécu psychologique et social de la surveillance communautaire des personnes confinées ainsi que de monter l´importance du feedback du travail de l´équipe psychosociale dans la gestion globale de la pandémie.

Ce retour d´expérience s´est fondé sur les rapports d´activité de l´équipe psychosociale, rédigés par les intervenants de terrain au retour des visites à domiciles des personnes confinées. Cette équipe était composée de trois personnes, toutes formées à l´écoute et aux techniques de prise en charge psychosociale des victimes dans des situations d´urgences: un psychiatre, un médecin et une assistante sociale. Ces comptes rendus journaliers relatent plusieurs faits notamment les modalités d´accueil de l´équipe sur les sites de confinement, le nombre de personnes confinées, les thèmes et sujets abordés lors des entretiens avec les personnes contacts, les difficultés rencontrées, les besoins exprimés par les confinés et la présence de symptômes psychosomatiques depuis leur confinement. Dans le cadre de ce travail, les auteurs reviennent sur le vécu de la quarantaine des personnes contacts à partir de l´inventaire des pensées, des affects, des réactions et craintes survenant au cours de leur confinement. L´évaluation des rapports d´activités de l´équipe psychosociale a pu relever plusieurs situations pesantes et stressantes pour les confinés. Il s´agit du vécu que les victimes ont d´une part par rapport à la maladie et d´autres parts par rapport à des situations de communication et d´intervention sociale liées au contexte de la riposte. Parmi ces situations nous pouvons noter:

**Difficultés socio familiales:** le confinement communautaire de certaines personnes était très mal vécu. Certains sujets contacts misent en quarantaine se sentaient rejetés et exclus par leur propre famille. Il s´agissait surtout de jeunes femmes actives qui vivaient dans des familles avec des conflits préexistants. La situation de la COVID-19 est venue s´ajouter à ces antécédents de conflits « je n´avais pas une très bonne relation avec ma famille, je n´ai pas de frères, ni de sœurs dans cette maison, mes parents sont décédés depuis longtemps et je vis ici indépendamment de ma volonté. J´allais tout le temps au travail pour éviter de me disputer avec les cousines, (...), maintenant, elles m´adressent plus la parole et ne rentrent plus dans ma chambre. ». Ayant vécu directement l´intervention des équipes de suivi quotidien, certaines familles de personnes confinées ont tendance à isoler les personnes contacts dans un endroit où elles recevaient les intervenants. Cette « surprotection » des familles des personnes contacts semble jouer un rôle dans la genèse de la souffrance psychologique des confinées.

**Situations de revendication et de régression:** différentes réactions régressives ont été notées chez certains sujets contacts confinés à domicile. Le travail psychosocial de terrain a relevé des infantilisations avec de multiples revendications des confinés par rapport à leur situation actuelle « vous nous avez enfermé ici sans nous avertir », sur les denrées alimentaires, sur le souci de la nourriture après le confinement, sur des dettes non honorées et sur le délai de la mise en observation pour certains. Des demandes ont été formulées. La plupart des personnes confinées sont dans des situations socioéconomiques précaires. La quasi-totalité de ces personnes vivent du fruit de leurs activités quotidiennes. Cette situation de confinement les fragilise et les expose d´avantage. Certains sujets contacts sollicitent un accompagnement financier pour l´après confinement.

**Manque d´empathie des équipes de suivi:** certains sujets contacts ont très difficilement vécu la visite journalière de l´équipe médicale chargée du suivi des contacts (prise de température et recherche de signes). Les agents de suivi sont venus chez eux sans pour autant respecter la confidentialité et sans qu´aucune communication préalable ne les y ait préparé. Certaines personnes avaient même développé des moyens pour raccourcir la visite en répondant quasi immédiat par la négation à leurs interrogatoires à la recherche de symptômes. Cet état des faits semble être un équivalent au refus de collaboration voire même à un déni de morbidité de cette pathologie.

**Manque de communication et partage de leurs identifiants:** plusieurs personnes contacts déplorent le manque de communication sur leur maladie et les moyens mis en place pour les soutenir sur le plan matériel. D´autres ont déplorés le nombre de coups de téléphones d´inconnus qu´ils reçoivent. Ils ont l´impression que leurs numéros ont été partagés et leurs vies étaient mises à nue. Les nombreux appels des journalistes qui leur demandent leurs états de santé. Tous ces facteurs renforcent la vulnérabilité psychique des victimes. Ils favorisent aussi la stigmatisation.

**Difficultés liées à la stigmatisation et discrimination:** la stigmatisation dans ces contextes d´urgence est une question essentielle dans le travail d´accompagnement psychosocial. Certains sujets contacts rapportent des attitudes de rejet et d´évitement. La mise en distance physique, créée par le confinement, a été le fait des voisins, d´amis et bien souvent de membres de leur famille chez certains cas contacts. Des personnes fuient les alentours des maisons des contacts tandis que certains des amis ont limité leurs relations sociales avec ces derniers ou ils ont carrément coupé tous liens amicaux. Dans le cercle familial, le rejet est moins affirmé, même si certains tiers manifestent une réserve. Ces attitudes ont entrainé le développement chez certains de conduites de défense par la crainte d´être considéré comme une menace: repli sur soi, retrait physique et social, évitement des espaces de sociabilité. Ces conduites inappropriées peuvent générer des sentiments de détresse et de solitude, voire même une auto stigmatisation et une culpabilité découlant en parti du déni et du refus de certaines personnes suivies [4]. Par rapport à ces différentes situations stressantes, des actions de régulation ont été entreprises par l´équipe de soutien psychosocial avec le Centre de Promotion et de Réinsertion Sociale (CPRS) de la Région pour un accompagnement social des victimes. Ainsi, tout au long du processus, l´équipe psychosociale a communiqué ces observations à la coordination nationale et aux équipes de prise en charge et de suivi pour une meilleure évaluation de la situation. Des entretiens individuels ont été effectués avec certains sujets contacts à la fin de leur mise en quarantaine. Ces débriefings individuels ont donné l´occasion de rassurer, de modérer d´éventuelles réactions de stress, d´aider certaines victimes à assumer et à intégrer la situation. Ils ont également permis le repérage des sujets à suivre dans le long terme mais aussi d´évaluer le dispositif en gros et de formaliser un suivi ultérieur en ligne.

## Conclusion

La prise en charge psychosociale des personnes affectées par la COVID-19 à Touba a permis d´identifier chez les victimes leurs difficultés émotionnelles et matérielles. Elle a aussi permis tant bien que mal de rompre l´isolement des personnes contacts. Certains avaient besoins de prise en charge psychologique plus structurée et ont été référés vers des psychiatres ou psychologues. Un soutien social en denrée alimentaire et en argent a été apporté à des familles confinées. Outre l´effet thérapeutique et de soutien apporté par l´équipe psychosociale, les informations recueillies ont permis aux différentes commissions impliquées d´avoir une autre vision du confinement avec ces répercussions psychosociales. Les informations collectées permettent d´assurer une régulation de la riposte avec des retours d´expériences nécessaires pour la modélisation et le réajustement des différentes interventions.

## Remerciements

Nous remercions le Centre des Opérations d´Urgence Sanitaire (COUS) du Ministère de la Santé et de l´Action Sociale (MSAS) de nous avoir donné l´opportunité d´aller sur le terrain pour participer à cette aide humanitaire et riche d´expérience.
